# GROMOS-RONS: A Force Field for Simulations of Reactive Oxygen and Nitrogen Species

**DOI:** 10.1021/acs.jpcb.5c01926

**Published:** 2025-05-29

**Authors:** Rodrigo M. Cordeiro

**Affiliations:** Centro de Ciências Naturais e Humanas, Universidade Federal do ABC, Avenida dos Estados 5001, Santo André, São Paulo CEP 09210-580, Brazil

## Abstract

Reactive oxygen and nitrogen species (RONS) play pivotal roles in biological and atmospheric systems, yet their transient nature challenges experimental study. Molecular dynamics (MD) simulations offer a powerful alternative, as long as reliable molecular mechanical models are available that accurately reproduce key physical properties of the simulated species. Correct partitioning behavior is crucial for biomolecular and atmospheric chemistry simulations, where RONS interactions at interfaces—such as phospholipid membranes and water–air boundaries—underpin essential processes. This study presents GROMOS-RONS, a force field for MD simulations of RONS and related compounds within the framework of the GROMOS 53A6 and 54A7 force field families. By integrating electronic structure calculations, thermodynamic integration, and equilibrium MD simulations, parameters were optimized to reproduce solvation free energies of various RONS in both water and hydrophobic media. In the case of ionic species, emphasis was placed on the correct hydration structure and ion-pairing tendencies. This force field provides a robust, validated tool for studying RONS dynamics and interactions across diverse scientific domains.

## Graphical abstract

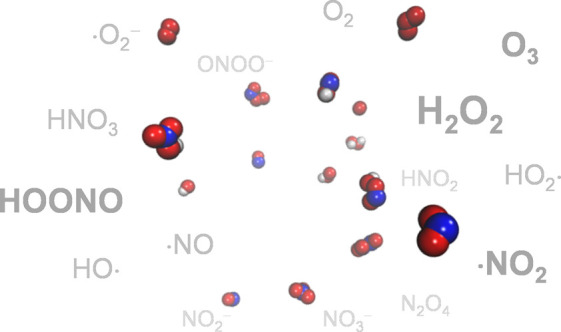

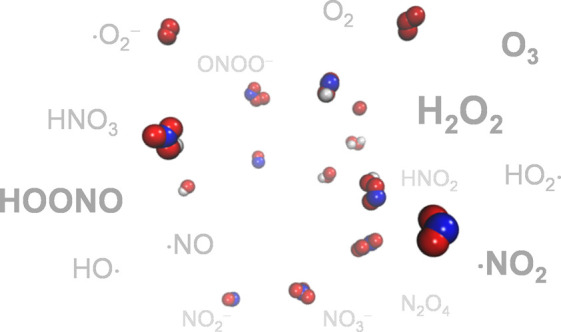

## 1 Introduction

Reactive oxygen and nitrogen species (RONS) encompass a class of molecules, ions, and radicals distinguished by their high chemical reactivity and transient nature. These species play critical roles across diverse domains, from biochemistry to atmospheric chemistry. In biological systems, RONS regulate redox signaling at low concentrations.^1^ However, their overproduction induces oxidative stress, contributing to pathological conditions such as aging and disease.^2^ In the atmosphere, RONS govern aerosol chemistry through interactions at water–air interfaces.^3^ Despite their importance, experimental techniques often lack the resolution to capture the rapid dynamics and nanoscale interactions of RONS within complex environments like interfaces, phospholipid membranes, and protein channels.

Molecular dynamics (MD) simulations have emerged as a powerful tool to address this gap. Over the past decade, our research has leveraged MD simulations to investigate RONS behavior, including their partitioning in phospholipid bilayers,^4–6^ transport through aquaporins,^7^ and dynamics at water–air interfaces.^8^ Although these studies have yielded well-validated force field parameters for RONS, the efforts remain scattered across multiple publications, resulting in a fragmented parameter set. Moreover, these parameters were primarily optimized to reproduce hydration free energies derived from reference Henry’s law constants.^9^ These experimental benchmarks, which quantify RONS solubility and partitioning behavior, are subject to continuous revision and updating as new measurements are made available.^10^

This study revisits prior parametrizations in light of updated solvation data, refining and consolidating past developments into a unified GROMOS-type force field for a variety of RONS, as depicted in Figure 1. More stable species that form as a result of RONS reactions were also included. Existing parameters from prior publications were cross-checked with updated solvation data, validated, and compiled into a user-friendly parameter library, named the GROMOS-RONS force field. This consolidation eliminates the need for users to compile parameters from multiple sources, reducing errors and ensuring consistent application across studies. New improvements were also introduced, including: *i*) an updated set of partial charges for the HO_2_· radical, more consistent with quantum chemistry charge distributions, settling differences between parameters in prior studies,^4,7^ while keeping agreement with experimental solvation free energies; *ii*) improved interaction parameters for ·O_2_^–^, resolving excessive ion clustering observed with the original parameters;^4^ *iii*) parametrization and inclusion of OONO^–^ as a new RONS; and *iv*) corrections of minor force field inconsistencies—such as swapping oxygen for nitrogen mass in one of the O_3_ atoms—and addition of missing improper dihedral terms to the original N_2_O_4_ model.^5^ While these corrections had minimal impact on previously reported solvation data, they enhanced force field rigor and correctness. Overall, this study provides a comprehensive account of the GROMOS-RONS force field development and validation, serving as a guiding tool for biomolecular and condensed-phase MD simulations of RONS.

**Figure 1:**
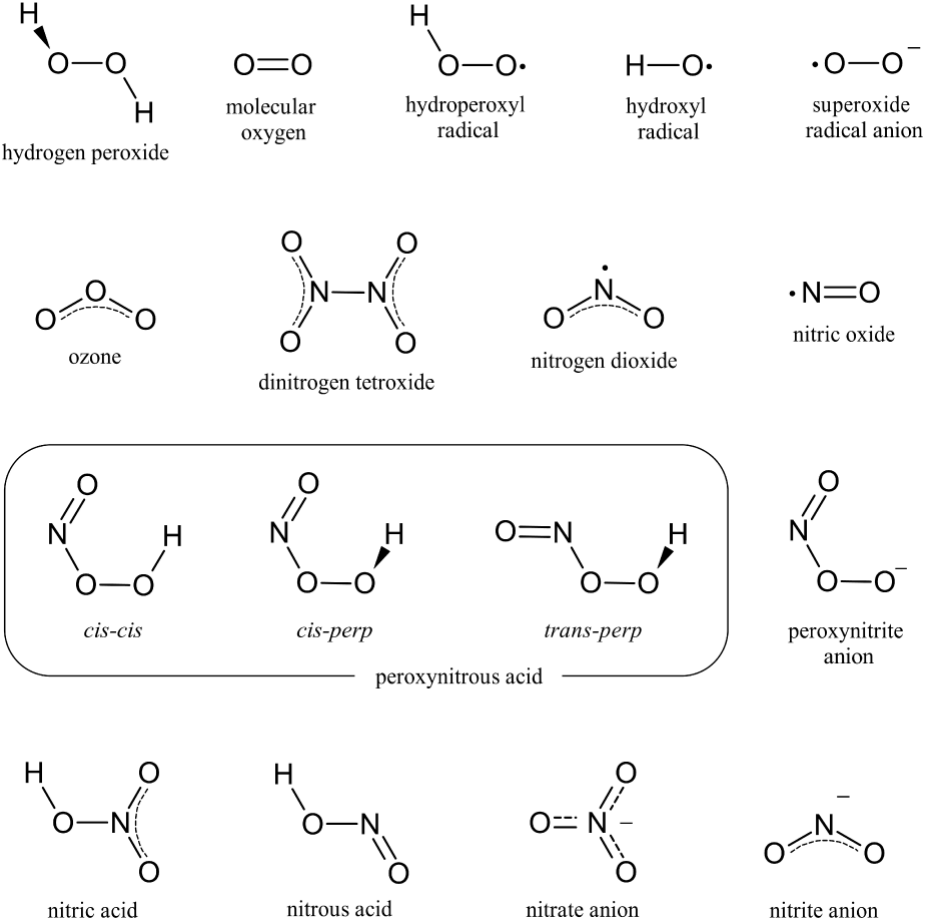
Chemical structures of the RONS and related molecules considered in force field parametrization.

## 2 Methods

### 2.1 Simulation Setup

The GROMACS software package^11,12^ was employed for MD simulations. Newton’s equations of motion were integrated using a time step of 2 fs. All bond lengths were constrained to their equilibrium values in order to eliminate high-frequency vibrational degrees of freedom and increase computational efficiency. Periodic boundary conditions were applied in all Cartesian directions. Lennard-Jones interactions were treated with a dual-range cutoff at 0.9 and 1.4 nm, including long-range dispersion corrections for energy and pressure. Electrostatic interactions were computed using the particle mesh Ewald (PME) method with a real-space cutoff of 0.9 nm. Condensed-phase simulations were performed in the isothermal–isobaric (*NPT*) ensemble at 298 K and 1 bar. Interatomic interactions were modeled based on the GROMOS 53A6 force field^13^ in conjunction with the SPC water model. Notably, our use of PME electrostatics and long-range dispersion corrections deviated from standard GROMOS settings. However, as demonstrated previously,^8^ these choices have a minor effect on pure-liquid properties and solvation energies.

### 2.2 Force Field Functional Form

The GROMOS 53A6 force field adopts a united-atom approach, wherein nonpolar hydrogen atoms (e.g., those bound to carbon) are implicitly included in the parameters of their bonded heavy atoms, enhancing computational efficiency for large biomolecular systems. Bonded interactions, which maintain molecular structure and geometry, consist of four distinct contributions: bond stretching, angle bending, improper dihedral bending (e.g., to preserve planarity of molecular fragments), and proper dihedral rotation. The potential energies associated with these terms are expressed as

1 $$V_{bond}=\frac{1}{4}K_{b}{\left(b^{2}-b_{0}^{2}\right)}^{2}$$

2 $$V_{angle}=\frac{1}{2}K_{\theta }{\left(\cos\theta -\cos\theta_{0}\right)}^{2}$$

3 $$V_{improper}=\frac{1}{2}K_{\xi }{\left(\xi -\xi_{0}\right)}^{2}$$

4 $$V_{proper}=\underset{i}{\sum }K_{\varphi i}\left[1+\cos\left(m_{i}\varphi -\varphi_{0i}\right)\right]$$

where *b* stands for the covalent bond length, θ for the angle between three consecutively bonded atoms, ξ for the improper dihedral angle, and φ for the proper dihedral angle defined by four consecutively bonded atoms. Parametrization involves determining the values of the force constants *K*, the reference bond lengths and angles (denoted by the subscript 0), and, for proper dihedrals, the multiplicity *m*. To enhance flexibility and capture complex dihedral profiles, the proper dihedral potential is formulated as a sum over terms with different multiplicities. Alternatively, a Ryckaert–Bellemans (RB) potential may be used:

5 $$V_{RB}=\overset{5}{\underset{i=0}{\sum }}C_{i}{\left[\cos\left(\varphi -180^{◦}\right)\right]}^{i}$$

As a side note, the bond stretching (eq 1) and angle bending (eq 2) potentials in GROMOS deviate from traditional harmonic functional forms. For computational efficiency, these potentials are intentionally anharmonic, yet they closely resemble their harmonic counterparts within the range of physically relevant energies.^13^

Nonbonded interactions arise from pairwise contributions between atoms that are not covalently linked or are beyond a specified neighbor exclusion limit. The nonbonded potential between atoms *i* and *j* comprises Lennard-Jones (LJ) and electrostatic Coulombic (C) terms:

6 $$V_{LJ}=\frac{C_{12}\left(i,j\right)}{r^{12}}-\frac{C_{6}\left(i,j\right)}{r^{6}}$$

7 $$V_{C}=\frac{q_{i}q_{j}}{4\pi \varepsilon_{0}r}$$

where *C*_12_ is the LJ repulsive parameter, *C*_6_ the attractive dispersion term, *r* the interatomic distance, *q* the atomic partial charge, and ε_0_ the dielectric permittivity of vacuum. The *C*_6_ parameters are tabulated for the self-interaction of each atom type. Geometric combination rules are then employed for interactions between different atom types, so that

8 $$C_{6}\left(i,j\right)=\sqrt{C_{6}\left(i,i\right)C_{6}\left(j,j\right)}$$

Similar rules apply for *C*_12_. However, up to three distinct *C*_12_(*i*,*i*) values are defined per atom type, which are then selected according to the specific pair-type combination. In practice, the value of *C*_12_(*i*,*j*) ends up being tabulated individually for each pair-type combination. The first and second nearest neighbors of an atom within a molecule are excluded from nonbonded interactions. When a proper dihedral potential is employed, third-neighbor (1–4) nonbonded interactions are included with modified *C*_12_ values. In contrast, when the RB dihedral potential was employed, 1–4 nonbonded interactions were excluded.

### 2.3 Parametrization Strategy

Interatomic interaction parameters were developed for all species represented in Figure 1. Bonded interactions were derived either from electronic structure calculations or experimental spectroscopy and crystallography data (cf. 4–8 and references therein). For complex molecules with multiple dihedrals, such as HOONO, dihedral parametrization proceeded as follows. The classical model was energy-minimized with all degrees of freedom unrestrained, except for dihedrals and bond lengths, which were fixed as rigid bonds, following the force field setup. Dihedral restraints were applied to maintain conformations matching those from reference quantum chemistry calculations. After minimization, dihedral restraints were removed, and single-point energy calculations were performed for the classical model without dihedral potentials. Classical dihedral potentials were then derived from the energy difference between the classical and quantum calculations. LJ parameters, representing new atom types, were developed to reproduce pure-liquid properties (i.e., density and heat of vaporization) of H_2_O_2_ and HNO_3_, the only species forming stable liquid phases at ambient temperature and pressure.^14,15^ Atom-centered partial charges were adjusted to match reference hydration free energies.^10^ Initial charge distributions were derived from electronic structure calculations using the Gaussian software.^16^ Calculations employed the B3LYP level of theory with either 6-31G­(d) or 6-311++G­(3df,3pd) basis sets and the CHelpG charge assignment scheme. These atom-centered partial charges served as the starting point for solvation free energy calculations via the thermodynamic integration (TI) method.^17^ Charges were iteratively scaled to account for solvent-induced polarization until agreement with reference hydration free energy data was achieved. In the case of gaseous species such as O_2_, O_3_, ·NO, ·NO_2_ and N_2_O_4_, for which solvation free energies in hydrocarbons have been experimentally measured,^18–23^ LJ parameters and partial charges were refined to accurately describe solvation in both aqueous and hydrocarbon media. For ionic species, atom types were selected and charges adjusted to reproduce hydration structures (radial distribution functions) from *ab initio* MD simulations from the literature,^24–28^ as well as experimentally measured solution densities^29^ and ion-pairing tendencies.^30^

### 2.4 Thermodynamic Integration

In the TI method, solvation free energies are computed by gradually decoupling the solute from its solvent environment through scaling all solute–solvent interactions to zero. A scaling parameter, λ, is systematically varied between 0 (full interactions) and 1 (no interactions) across a series of MD simulations. The solvation free energy is then obtained by integrating the ensemble-averaged derivative of the total Hamiltonian (*H*) with respect to λ:

9 $$\Delta G_{solv}=-\left[{\int_{0}^{1}\left\langle \frac{\partial H\left(\lambda \right)}{\partial \lambda }\right\rangle }_{\lambda }^{C}d\lambda +{\int_{0}^{1}\left\langle \frac{\partial H\left(\lambda \right)}{\partial \lambda }\right\rangle }_{\lambda }^{LJ}d\lambda \right]$$

To prevent charge clashes, Coulomb interactions were decoupled first, followed by LJ interactions. We employed 21 and 51 intermediate states for Coulomb and LJ interactions, respectively, ensuring accurate numerical integration of eq 9. To achieve proper thermalization of the solute in the fully decoupled state, Langevin stochastic dynamics was applied with a friction constant of 1 ps^–1^. A single solute molecule was placed in a cubic solvent box with an edge length of ∼3 nm. Each intermediate state underwent a 600 ps equilibration phase, followed by 5 ns of data acquisition.

### 2.5 Equilibrium Simulations

Molecular dynamics simulations of the pure liquid state were conducted in the *NPT* ensemble at 1 bar and 298 K. Each simulation consisted of an equilibration phase and a sampling phase, both lasting 5 ns. Ensemble averages of the liquid density (ρ) and the interatomic potential energy (*U*) were obtained from the sampling phase. To calculate the heat of vaporization (Δ*H*_vap_), simulations of isolated molecules in vacuum were conducted to model the gaseous state. The heat of vaporization was determined using the relation:

10 $$\Delta H_{vap}=U_{gas}-U_{liq}+RT$$

where *R* is the ideal gas constant and *T* is the absolute temperature. Simulations of ionic species in aqueous solutions were performed across a range of concentrations, from the infinitely dilute regime (i.e. a single ion pair in a cubic water box with ∼3 nm sides) to a concentration of ∼6.5 mol/L. Charge neutrality was maintained by including Na^+^ counterions. These systems were equilibrated in the *NPT* ensemble for 5 ns, followed by a 25 ns data acquisition phase. Radial distribution functions (RDFs) were computed to characterize the distribution of hydration waters around the ions. The positions of the minima following the first RDF peaks were used as distance cutoffs to determine ion-pair interactions.

## 3 Results

### 3.1 Overview

The development of the GROMOS-RONS force field is documented here. This subsection provides a general overview of the parametrization and validation process, while detailed analyses of individual species are reserved for the following subsections. The newly developed atom types are summarized in Table 1, with their corresponding LJ self-interaction parameters detailed in Tables 2 and 3. The specific pair interactions that were probed during parametrization in different solvent environments are outlined in Table 4. The GROMOS database includes a broader range of atom types whose interactions with the new types were not directly assessed. Instead, these interactions were derived using combination rules (cf. eq 8), with the *C*_12_ parameter selection based on Table 5. As stated before, the force field was validated through simulations of pure liquid properties and solvation free energies. In Tables 6–8 these results are benchmarked against reference data. Solvation free energies provide a robust foundation for force field parametrization, as they govern partition phenomena, which are typically the focus of condensed-phase RONS simulations. While solvation enthalpies can be computed from interaction energies, accurate hydration free energies do not always correlate with accurate hydration enthalpies.^31^ Inaccuracies in enthalpic and entropic contributions may balance to produce accurate solvation free energies. This issue is not solely a limitation of the parametrized solute but also of the solvent model underlying the force field. This underscores the caveat that no force field is universally optimal for all properties, requiring a balanced compromise and focus on the most relevant properties. The complete set of force field parameters is compiled in Table 9, with the associated files provided as Supporting Information for reproducibility and further application. Note that bond stretching constants were left out of the parametrization process because the model is intended for use with bond constraints. Values of bond stretching constants in the molecular topology files are included as mere placeholders. For reference, Table 10 summarizes the partial charges from quantum mechanical calculations, used as starting points for solvation free energy calculations. For molecules with multiple conformers, conformer-specific charge distributions were computed separately and averaged to produce a unified molecular mechanical model. Data are presented only for the largest molecules. For small species, such as HO·, the empirical charge optimization protocol in GROMOS-RONS rendered quantum mechanical charge calculations unnecessary due to trivial charge distributions (e.g., two atoms with equal but opposite charges). An empirical, trial-and-error charge optimization approach was used, acting on a single variable that could be arbitrarily set and iteratively refined. This approach also applied to larger, symmetrical molecules like H_2_O_2_.

**Table 1:** Newly Developed Atom Types

Atom type	Mass (a.m.u)	Description
OP	15.999	OH-group oxygen in hydrogen peroxide, oxyradicals and nitrogen oxyacids
OO	15.999	oxygen in molecular oxygen and nitric oxide
OQ	15.999	oxygen in ozone, nitrogen oxides and nitrogen oxyacids
NQ	14.007	nitrogen in nitrogen oxides and nitrogen oxyacids

**Table 2:** LJ Parameters for Nonbonded Interactions

		[*C*_12_(*i*,*i*)]^1/2^ 10^–3^ (kJ·mol^–1^·nm^12^)^1/2^		
Atom type	[*C*_6_(*i*,*i*)]^1/2^ (kJ·mol^–1^·nm^6^)^1/2^	1	2	3
OP	0.04756	1.334	1.334	—
OO	0.018963	0.33329	—	—
OQ	0.035765	1.000	0.834	1.784
NQ	0.035765	1.000	0.834	—

**Table 3:** LJ Parameters for Third-Neighbor 1–4 Interactions

Atom type	[*C*_6_(*i*,*i*)]^1/2^ (kJ·mol^–1^·nm^6^)^1/2^	[*C*_12_(*i*,*i*)]^1/2^ 10^–3^ (kJ·mol^–1^·nm^12^)^1/2^
OP	0.04756	1.334
OQ	0.035765	0.834

**Table 4:** Properties and Species Involved in Development and Validation of Nonbonded Interactions

Interaction		Validating properties^a^					Validating species										
*i*	*j*	ρ	Δ*H*_vap_	Δ*G*_w_	Δ*G*_ch_	Δ*G*_d_	H_2_O_2_	O_2_	HO_2_·	HO·	O_3_	N_2_O_4_	·NO_2_	·NO	HOONO	HNO_3_	HNO_2_
OP	OP	×	×				×									×	
OP	OW			×			×		×	×					×	×	×
OO	OW			×				×						×			
OO	CH2r				×			×						×			
OQ	OW			×							×	×	×		×	×	×
OQ	CH2					×					×	×	×				
OQ	CH3					×					×	×	×				
NQ	OW			×								×	×	×	×	×	×
NQ	CH2					×						×	×				
NQ	CH3					×						×	×				
NQ	CH2r				×									×			
OP	OQ	×	×													×	
OP	NQ	×	×													×	
OQ	OQ	×	×													×	
OQ	NQ	×	×													×	
NQ	NQ	×	×													×	

a Pure-liquid density (ρ), heat of vaporization (Δ*H*_vap_), hydration free energy (Δ*G*_w_), solvation free energy in cyclohexane (Δ*G*_ch_), solvation free energy in *n*-decane (Δ*G*_d_).

**Table 5:** Selection of LJ Repulsive *C*_12_ Parameters^a^

Selection of *C*_12_(*i*,*i*)					Selection of *C*_12_(*j*,*j*)				
	atom *j*					atom *j*			
atom *i*	OP	OO	OQ	NQ	atom *i*	OP	OO	OQ	NQ
O	2	1	1	2	O	2	1	1	2
OM	2	1	1	2	OM	2	1	1	2
OA	2	1	2	2	OA	2	1	2	2
OE	2	1	1	2	OE	2	1	1	2
OW	2	1	2	2	OW	2	1	2	2
N	2	1	2	2	N	2	1	2	2
NT	2	1	2	2	NT	2	1	2	2
NL	2	1	2	2	NL	2	1	2	2
NR	2	1	2	2	NR	2	1	2	2
NZ	2	1	2	2	NZ	2	1	2	2
NE	2	1	2	2	NE	2	1	2	2
C	1	1	1	1	C	1	1	1	1
CH0	1	1	1	1	CH0	1	1	1	1
CH1	1	1	1	1	CH1	1	1	1	1
CH2	1	1	1	1	CH2	1	1	1	1
CH3	1	1	1	1	CH3	1	1	1	1
CH4	1	1	1	1	CH4	1	1	1	1
CH2r	1	1	1	1	CH2r	1	1	1	1
CR1	1	1	1	1	CR1	1	1	1	1
HC	1	1	1	1	HC	1	1	1	1
H	1	1	1	1	H	1	1	1	1
DUM	1	1	1	1	DUM	1	1	1	1
S	1	1	1	1	S	1	1	1	1
CU1+	2	1	2	2	CU1+	2	1	2	2
CU2+	2	1	2	2	CU2+	2	1	2	2
FE	2	1	2	2	FE	2	1	2	2
ZN2+	2	1	2	2	ZN2+	2	1	2	2
MG2+	2	1	2	2	MG2+	2	1	2	2
CA2+	2	1	2	2	CA2+	2	1	2	2
P	1	1	1	1	P	2	1	2	2
AR	1	1	1	1	AR	1	1	1	1
F	2	1	1	2	F	2	1	1	2
CL	1	1	1	1	CL	2	1	1	2
BR	1	1	1	1	BR	2	1	1	2
CMet	1	1	1	1	CMet	1	1	1	1
OMet	2	1	2	2	OMet	2	1	2	2
NA+	2	1	2	2	NA+	2	1	2	2
CL–	2	1	1	2	CL–	2	1	1	2
CChl	1	1	1	1	CChl	1	1	1	1
CLChl	1	1	1	1	CLChl	1	1	1	1
HChl	1	1	1	1	HChl	1	1	1	1
SDmso	1	1	1	1	SDmso	1	1	1	1
CDmso	1	1	1	1	CDmso	1	1	1	1
ODmso	2	1	1	2	ODmso	2	1	1	2
CCl4	1	1	1	1	CCl4	1	1	1	1
CLCl4	1	1	1	1	CLCl4	1	1	1	1
FTfe	2	1	1	2	FTfe	2	1	1	2
CTfe	1	1	1	1	CTfe	1	1	1	1
CHTfe	1	1	1	1	CHTfe	1	1	1	1
OTfe	2	1	1	2	OTfe	2	1	1	2
CUrea	1	1	1	1	CUrea	1	1	1	1
OUrea	2	1	1	2	OUrea	2	1	1	2
NUrea	2	1	2	2	NUrea	2	1	2	2
SI	1	1	1	1	SI	2	1	2	2
OML	2	1	1	2	OML	2	1	1	2
CH3L	1	1	1	1	CH3L	1	1	1	1
OP	2	1	2	2	OP	2	1	3	2
OO	1	1	1	1	OO	1	1	1	1
OQ	3	1	1	2	OQ	2	1	1	2
NQ	2	1	2	2	NQ	2	1	2	2

a For example, the *C*_12_(*i*,*j*) parameter for the OP–OQ interaction results from the combination of the *C*_12_(*i*,*i*)-2 parameter for OP with the *C*_12_(*j*,*j*)-3 parameter for OQ.

**Table 6:** Pure-Liquid Properties of Selected RONS

	ρ (g/cm^3^)		Δ*H*_vap_ (kJ/mol)	
Substance	This work	Reference data^a^	This work	Reference data^a^
H_2_O_2_	1.38	1.44	57.3	51.6
HNO_3_	1.39	1.50	41.0	39.1

a Experimental data from the literature.^14,15^

**Table 7:** Hydration Free Energies of RONS

	Δ*G*_w_ (kJ/mol)	
Species	This work	Reference data^a^
H_2_O_2_	–36.8	-36.2 ± 0.5
O_2_	8.3	8.6 ± 0.2
HO_2_·	–28.9	-27 ± 3
HO·	–17.0	-17 ± 2
O_3_	3.0	3.2 ± 0.5
N_2_O_4_	–8.5	-9.4 ± 0.9
·NO_2_	1.6	2.6 ± 1.3
·NO	7.0	7.6 ± 0.5
*trans*-*perp*-HOONO	–22.1	
*cis*-*cis*-HOONO	–21.0	
HOONO	–21.0^b^	–21^c^
HNO_3_	–37.8	-39 ± 3
*trans*-HNO_2_	–18.0	
*cis*-HNO_2_	–11.2	
HNO_2_	–17.1^d^	-17.5 ± 0.2

a Unless otherwise noted, reference data are averages from a comprehensive database of Henry’s law constants derived from experimental measurements and thermodynamic considerations.^10^

b Δ*G*_w_ of HOONO considered to be the same as of the *cis*-*cis* conformer, given its greater stability and prevalence.

c Derived from the standard hydration free energy of HOONO^40^ following the procedure outlined in ref. 5

d Weighted average between conformers according to eq 11.

**Table 8:** Solvation Free Energies of RONS in Hydrocarbons

		Δ*G*_solv_ (kJ/mol)		
Species	Solvent	This work	Reference data	Ref.
O_2_	cyclohexane	3.0	3.2	^18,19^
O_3_	*n*-decane	–0.6	–0.6^a^	^20^
N_2_O_4_	*n*-decane	–7.1	–7.3^a^	^21^
·NO_2_	*n*-decane	–1.0	–0.9^a^	^22^
·NO	cyclohexane	2.5	2.1	^23^

a In the original references, decane was reported as the solvent without specification of its isomeric form (e.g., *n*-decane or other isomers).

**Table 9:** Force Field Parameters for RONS^a^

Species	Ref.	Interaction	Force field parameters
H_2_O_2_	^4^	O	type OP; *q* = – 0.42 *e*
		H	type H; *q* = +0.42 *e*
		O—O	*b*_0_ = 0.1443 nm
		O—H	*b*_0_ = 0.0981 nm
		O—O—H	θ_0_ = 100.36°; *K*_*θ*_ = 524.48 kJ/mol
		H—O—O—H	*C*_0_ = 1.99544235 kJ/mol; *C*_1_ = – 10.72695 kJ/mol;
			*C*_2_ = 15.22612 kJ/mol; *C*_3_ = – 2.24420 kJ/mol;
			*C*_4_ = 0.25839 kJ/mol; *C*_5_ = 0
O_2_	^4^	O	type OO; *q* = 0
		O—O	*b*_0_ = 0.1210 nm
HO_2_·	^7^	O	type O; *q* = – 0.17 *e*
		O(H)	type OP; *q* = – 0.293 *e*
		H	type H; *q* = +0.463 *e*
		O—O	*b*_0_ = 0.1332 nm
		O—H	*b*_0_ = 0.0981 nm
		O—O—H	θ_0_ = 105.08°; *K*_*θ*_ = 524.48 kJ/mol
HO·	^4^	O	type OP; *q* = – 0.436 *e*
		H	type H; *q* = +0.436 *e*
		O—H	*b*_0_ = 0.0981 nm
O_3_	^5^	O	type OQ; *q* = – 0.137 *e*
		O (central)	type OQ; *q* = +0.274 *e*
		O—O	*b*_0_ = 0.1264 nm
		O—O—O	θ_0_ = 117.92°; *K*_*θ*_ = 610.00 kJ/mol
N_2_O_4_	^5^	O	type OQ; *q* = – 0.292 *e*
		N	type NQ; *q* = +0.584 *e*
		O—N	*b*_0_ = 0.1196 nm
		N—N	*b*_0_ = 0.1783 nm
		O—N—O	θ_0_ = 134.72°; *K*_*θ*_ = 1866.89 kJ/mol
		O—N—N	θ_0_ = 112.64°; *K*_*θ*_ = 530.00 kJ/mol
		O—N—N—O	*C*_0_ = 27.878; *C*_1_ = 0; *C*_2_ = – 27.878 kJ/mol; *C*_3_ = 0; *C*_4_ = 0; C_5_ = 0
		N—N—O—O	ξ_0_ = 0.00°; *K*_*ξ*_ = 502.08 kJ/mol
·NO_2_	^5^	O	type OQ; *q* = – 0.189 *e*
		N	type NQ; *q* = +0.378 *e*
		O—N	*b*_0_ = 0.1199 nm
		O—N—O	θ_0_ = 133.89°; *K*_*θ*_ = 1866.89 kJ/mol
·NO	^6^	O	type OO; *q* = +0.02 *e*
		N	type NQ; *q* = – 0.02 *e*
		O—N	*b*_0_ = 0.1151 nm
HOONO	^5^	O	type OQ; *q* = – 0.126 *e*
		N	type NQ; *q* = +0.098 *e*
		O (central)	type OQ; *q* = – 0.014 *e*
		O(H)	type OP; *q* = – 0.407 e
		H	type H; *q* = +0.449 *e*
		N—O	*b*_0_ = 0.1194 nm
		N—O(O)	*b*_0_ = 0.1453 nm
		O—O	*b*_0_ = 0.1444 nm
		O—H	*b*_0_ = 0.0984 nm
		O—N—O	θ_0_ = 111.20°; *K*_*θ*_ = 1269.51 kJ/mol
		N—O—O	θ_0_ = 104.70°; *K*_*θ*_ = 640.00 kJ/mol
		O—O—H	θ_0_ = 100.57°; *K*_*θ*_ = 524.48 kJ/mol
		O—N—O—O	*m*_1_ = 1; φ_01_ = 0; *K*_φ1_ = – 15.2364 kJ/mol
			*m*_1_ = 2; φ_02_ = 0; *K*_φ2_ = – 26.4524 kJ/mol
		N—O—O—H	*m*_1_ = 1; φ_01_ = 0; *K*_φ1_ = 0.691523 kJ/mol
			*m*_2_ = 1; φ_02_ = 0; *K*_φ2_ = 3.90789 kJ/mol
			*m*_3_ = 1; φ_03_ = 0; *K*_φ3_ = – 1.2377 kJ/mol
			*m*_4_ = 1; φ_04_ = 0; *K*_φ4_ = – 0.606849 kJ/mol
HNO_3_	^8^	O	type OQ; *q* = – 0.445 *e*
		N	type NQ; *q* = +0.964 *e*
		O(H)	type OP; *q* = – 0.571 *e*
		H	type H; *q* = +0.497 *e*
		N—O	*b*_0_ = 0.1204 nm
		N—O(H)	*b*_0_ = 0.1405 nm
		O—H	*b*_0_ = 0.0961 nm
		O—N—O	θ_0_ = 130.22°; *K*_*θ*_ = 3772.45 kJ/mol
		O—N—O(H)	θ_0_ = 114.90°; *K*_*θ*_ = 2072.32 kJ/mol
		N—O—H	θ_0_ = 102.22°; *K*_*θ*_ = 556.51 kJ/mol
		O—N—O—H	*C*_0_ = 32.758724 kJ/mol; *C*_1_ = 0;
			*C*_2_ = −32.758724 kJ/mol; *C*_3_ = 0; *C*_4_ = 0; *C*_5_ = 0
		N—O—O—O	ξ_0_ = 0.00°; *K*_*ξ*_ = 502.08 kJ/mol
HNO_2_	^8^	O	OQ; *q* = – 0.121 *e*
		N	NQ; *q* = +0.093 *e*
		O(H)	OP; *q* = – 0.371 *e*
		H	H; *q* = +0.399 *e*
		N—O	*b*_0_ = 0.1201 nm
		N—O(H)	*b*_0_ = 0.1404 nm
		O—H	*b*_0_ = 0.0985 nm
		O—N—O	θ_0_ = 111.58°; *K*_*θ*_ = 1702.65 kJ/mol
		N—O—H	θ_0_ = 102.98°; *K*_*θ*_ = 571.74 kJ/mol
		O—N—O—H	*C*_0_ = 38.651923 kJ/mol; *C*_1_ = −5.895607 kJ/mol;
			*C*_2_ = – 37.873698 kJ/mol; *C*_3_ = 5.117382 kJ/mol;
			*C*_4_ = 0; *C*_5_ = 0
NO_3_^–^	^8^	N	type NR; *q* = +0.65 *e*
		O	type OM; *q* = – 0.55 *e*
		N—O	*b*_0_ = 0.1268 nm
		O—N—O	θ_0_ = 120.00°; *K*_*θ*_ = 1172.35 kJ/mol
		N—O—O—O	ξ_0_ = 0.00°; *K*_*ξ*_ = 502.08 kJ/mol
NO_2_^–^	^8^	N	type NR; *q* = +0.4 *e*
		O	type OM; *q* = – 0.7 *e*
		N—O	*b*_0_ = 0.1254 nm
		O—N—O	θ_0_ = 116.80°; *K*_*θ*_ = 1250.72 kJ/mol
·O_2_^–^	—	O	type OM; *q* = – 0.5 *e*
		O—O	*b*_0_ = 0.1280 nm
ONOO^–^	—	O(N)	type OQ; *q* = – 0.335 *e*
		N	type NQ; *q* – 0.064 *e*
		O (central)	type OQ; *q* = +0.013 *e*
		O	type OM; *q* = – 0.614 *e*
		N—O	*b*_0_ = 0.1194 nm
		N—O(O)	*b*_0_ = 0.1453 nm
		O—O	*b*_0_ = 0.1444 nm
		O—N—O	θ_0_ = 116.80°; *K*_θ_ = 1250.72 kJ/mol
		N—O—O	θ_0_ = 115.96°; *K*_θ_ = 740.69 kJ/mol
		O—N—O—O	*m*_2_ = 1_;_ φ_01_ = 0; *K*_*φ1*_ = – 7.5 kJ/mol
			*m*_2_*=*2*; φ*_02_ = 0; *K*_*φ2*_ = – 42.3 kJ/mol

a To be employed along with bond length constraints. Use of GROMOS-type dihedral potentials (eq 4) is indicated by specification of parameters *m*, φ_0_, and *K*_*φ*_, while use of RB dihedral potentials (eq 5) is indicated by definition of parameters *C*_0_ to *C*_5_.

**Table 10:** Initial Partial Charges from Electronic Structure Calculations^a^

Molecule	Atom	Partial charge (*e*)	Basis set
HO_2_·	O	–0.143	6-31G(d)
	O(H)	–0.247	
	H	0.390	
O_3_	O	–0.117	6-31G(d)
	O (central)	0.234	
·NO	O	0.012	6-31G(d)
	N	–0.012	
HOONO^b^	O	–0.141/-0.082	6-31G(d)
	N	0.071/0.102	
	O (central)	0.004/-0.029	
	O(H)	–0.335/-0.381	
	H	0.401/0.390	
HNO_3_	O^c^	–0.317/-0.392	6-311++G(3df,3pd)
	N	0.768	
	O(H)	–0.455	
	H	0.396	
HNO_2_ ^d^	O	–0.151/-0.067	6-311++G(3df,3pd)
	N	0.126/0.041	
	O(H)	–0.362/-0.304	
	H	0.387/0.330	
NO_3_^–^	N	1.067	6-311++G(3df,3pd)
	O	–0.689	
NO_2_^–^	N	–0.112	6-311++G(3df,3pd)
	O	–0.444	
ONOO^–^ ^d^	O(N)	–0.249/-0.420	6-311++G(3df,3pd)
	N	–0.156/0.027	
	O (central)	–0.046/0.072	
	O	–0.549/-0.679	

a Obtained using the B3LYP level of theory and CHelpG charge fitting.

b Charges reported for different conformers (*cis-cis*/*trans-perp*).

c Terminal O atoms were considered with equal charges in the molecular mechanical model.

d Charges reported for different conformers (*cis*/*trans*).

### 3.2 Hydrogen Peroxide

The most stable conformation of hydrogen peroxide (H_2_O_2_) is a skewed structure with a dihedral angle of ∼112°.^32^ Its torsional energy profile is characterized by a shallow barrier at the *trans* conformation, between its two equivalent minima, and a higher barrier at the fully staggered state. These features were captured by using the appropriate RB dihedral potential (Table 9). Initial attempts to reproduce pure liquid properties of H_2_O_2_ with the standard GROMOS hydroxyl-oxygen atom type (OA) were unsuccessful, prompting the development of a new peroxide-oxygen atom type (OP) for use in conjunction with polar hydrogen (type H) (Table 2). The *C*_12_ LJ parameters for OP were selected in analogy to the interaction matrix established for the standard OA atom type (Table 5).^13^ In addition to the development of the OP atom type, partial atomic charges were tuned to achieve pure liquid properties (Table 6) and hydration free energy (Table 7) in close agreement with experimental data. It is worth noting that nonbonded interaction parameters are interdependent and should be evaluated as a consistent set within a given force field. Differences in parametrization strategies often balance to yield comparable results. For example, in earlier MD simulations by Vácha et al., oxygen atoms in H_2_O_2_ were represented by the OW atom type from water, combined with a more polarized set of partial charges.^33^ This led to a relatively accurate hydration free energy of H_2_O_2_, although its pure-liquid properties were not assessed. Similarly, successful H_2_O_2_ models have been developed for different force field families, based on distinct parametrization strategies and models for water as solvent.^34^ In GROMOS-RONS, parameters were optimized for consistency within the classical GROMOS framework, and all parameters are provided (Tables 1, 2, 3, 5, and 9) for further analysis. Figure 2 demonstrates proper convergence of the free energy calculations by the TI method within the simulated time scale. Since Coulomb and LJ interactions were decoupled separately, their individual contributions could be evaluated from numerical integration of the curves in Figure 2. For H_2_O_2_, we found that LJ interactions contributed +6.6 kJ/mol to the total hydration free energy of −36.8 kJ/mol, reflecting an energy penalty due to solute inclusion and formation of the solvent cage. In contrast, the Coulomb contribution of −43.4 kJ/mol dominated solvation, driven by strong solute–solvent hydrogen bonds. The relative contributions of Coulomb and LJ terms are expected to vary depending on the specific solute and solvent under consideration.

**Figure 2:**
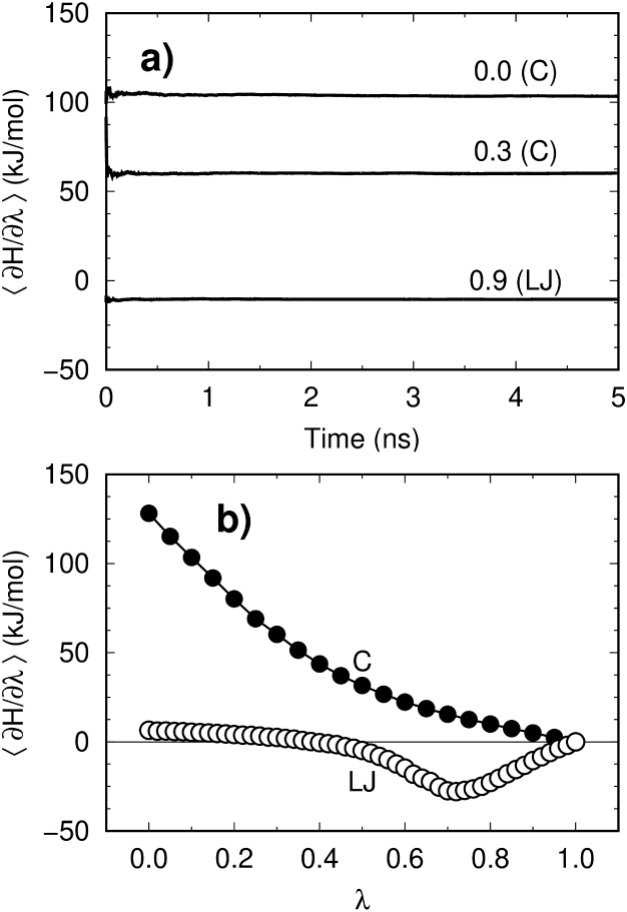
Convergence of TI simulations for computation of H_2_O_2_ hydration free energy. (a) Time-dependent running averages and (b) final ensemble-average values of the derivative of the total Hamiltonian with respect to the scaling parameter λ. Numbers above the lines indicate the λ values corresponding to different states of Coulomb and LJ decoupling.

### 3.3 Molecular Oxygen

The development of a molecular mechanical model for oxygen (O_2_) poses challenges due to the simplicity of a GROMOS-type O_2_ model and its limited number of adjustable parameters. Without any virtual interaction sites to represent quadrupole moments, the symmetry of the two oxygen atoms requires that atom-centered partial charges are set to zero. Similarly, the united-atom representation of GROMOS hydrocarbons lacks partial charges, causing O_2_ solvation free energies in hydrocarbons to depend exclusively on LJ parameters. With charges fixed at zero, LJ parameters must also implicitly account for polarization effects of O_2_ in water. Incorporating classical Drude oscillators into molecular mechanical models effectively describes induced polarization.^35^ However, for greater simplicity, the O_2_ model adhered strictly to usual GROMOS-type interactions, leaving out Drude oscillators and virtual interaction sites for polarizability and multipole effects, respectively. To address these constraints, a new atom type, OO, was developed (Table 2). The LJ interaction parameters of the OO atom type deviated from other oxygen atom types, due to implicit incorporation of additional effects such as solvent-induced polarization and higher-order electrostatic effects. Parameterization of the OO atom type relied on its interactions with the standard GROMOS water-oxygen type (OW) and the cyclic hydrocarbon CH2r type (Table 4). The *C*_12_ LJ interaction matrix was established in analogy to the standard GROMOS argon atom (AR) (Table 5). The resulting model (Table 9) was capable of a balanced description of O_2_ solvation in both water (Table 7) and cyclohexane (Table 8).

### 3.4 Polar Oxyradicals

Molecular mechanical models for the hydroperoxyl (HO_2_·) and hydroxyl (HO·) radicals were constructed using the same peroxide-oxygen atom type (OP) developed for H_2_O_2_ (Table 9). Partial charges were adjusted for an accurate description of hydration free energies (Table 7).

### 3.5 Ozone and Nitrogen Oxides

New atom types, OQ and NQ, were developed to represent oxygen and nitrogen atoms, respectively, in ozone (O_3_), dinitrogen tetroxide (N_2_O_4_), and nitrogen dioxide (·NO_2_). LJ parameters (Table 2) were optimized to simultaneously reproduce the solvation free energies of all three species in hydrocarbon media (Table 8), while partial charges were adjusted to match their hydration free energies (Table 7). The *C*_12_ interaction matrix was established in analogy to the standard GROMOS atom types O for OQ, and NR for NQ (Table 5). For nitric oxide (·NO), solvation in both cyclohexane and water was satisfactorily described by simply combining atom types NQ and OO, with adjustments limited to partial charges. The resulting molecular mechanical models (Table 9) aligned with the expected molecular geometries. In the case of N_2_O_4_, a high-barrier RB dihedral potential^36^ was implemented alongside improper dihedrals to maintain planarity.

### 3.6 Peroxynitrous Acid

The molecular mechanical model for peroxynitrous acid (HOONO) was constructed using the previously developed atom types OP, OQ, and NQ. Intramolecular 1–4 interactions between OP and OQ were specified as in Table 3. The interplay between the O–N–O–O and N–O–O–H dihedrals gives rise to the conformers represented in Figure 1. Torsional potentials for the O–N–O–O and N–O–O–H dihedrals were calibrated against quantum chemistry reference data from the literature, obtained at the MP2 level of theory with the 6-31lG­(2df,2p) basis set.^37^ As shown in Figure 3 and Table 11, the molecular mechanical model captured key structural features of HOONO, despite minor deviations from reference geometries. Such deviations stem at least in part from the inability of the fully classical model to reproduce the coupling between the torsional degrees of freedom of HOONO and the central N–O bond length.^37–39^ In the gas phase, the *cis*-*cis* conformer of HOONO is the most prevalent, with its stability driven by an intramolecular hydrogen bond. In aqueous solution, competing interactions with water and the flexibility of the N–O–O–H dihedral allow exploration of the *cis*-*perp* conformation. Regarding the O–N–O–O dihedral, its *cis* state predominates in aqueous solution and is separated from the *trans* state by a large energy barrier. In multinanosecond simulations, *cis*-*trans* isomerization of O–N–O–O is a low probability event, enabling hydration free energies to be computed separately for each conformer, despite their shared molecular mechanical model (Table 9). The hydration free energies calculated for the *trans*-*perp* and *cis*-*cis* conformers were very similar and consistent with reference data (Table 7).^40^

**Figure 3:**
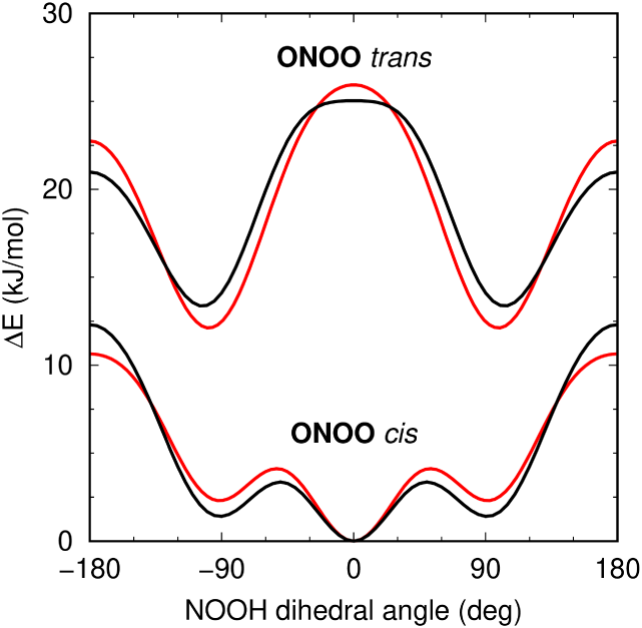
Energy profiles for rotation of the N–O–O–H dihedral angle in gas-phase HOONO at *cis*- and *trans*-conformations of the O–N–O–O dihedral. Results from the classical GROMOS-RONS force field (black) are compared with *ab initio* data (red) from the literature.^37^

**Table 11:** Geometries and Energies of HOONO Conformers^a^

	Bond lengths (nm)				Angles (°)			Dihedrals (°)		
Conformer	NO (terminal)	NO (central)	OO	OH	ONO	NOO	OOH	ONOO	NOOH	Δ*E* (kJ/mol)
*cis–cis*	**1.190**	**1.450**	**1.440**	**0.980**	**114.6**	**112.4**	**99.8**	**0.0**	**0**	**0**
	(1.211)	(1.386)	(1.445)	(0.993)	(114.3)	(113.6)	(100.6)	(0)	(0)	(0)
cis-gauche	**1.190**	**1.450**	**1.440**	**0.980**	**114.7**	**112.7**	**100.2**	**–2.5**	**50**	**3.4**
	(1.197)	(1.430)	(1.443)	(0.984)	(114.0)	(111.9)	(102.0)	(−7.5)	(53.9)	(4.2)
*cis-perp*	**1.190**	**1.450**	**1.440**	**0.980**	**114.9**	**113.1**	**100.6**	**–2.1**	**90**	**1.4**
	(1.182)	(1.491)	(1.440)	(0.980)	(113.9)	(109.7)	(101.0)	(−5.1)	(92.0)	(2.5)
*cis–trans*	**1.190**	**1.450**	**1.440**	**0.980**	**115.0**	**113.3**	**100.8**	**0.0**	**180**	**12.3**
	(1.191)	(1.427)	(1.472)	(0.980)	(114.8)	(108.5)	(95.6)	(0)	(180)	(10.5)
*perp–perp*	**1.190**	**1.450**	**1.440**	**0.980**	**111.5**	**106.0**	**100.8**	**95**	**100.5**	**55.1**
	(1.163)	(1.677)	(1.458)	(0.978)	(109.5)	(96.4)	(99.7)	(81.9)	(100.6)	(52.3)
*trans–cis*	**1.190**	**1.450**	**1.440**	**0.980**	**111.3**	**105.9**	**101.3**	**179.7**	**0**	**25.0**
	(1.194)	(1.433)	(1.459)	(0.986)	(110.3)	(106.1)	(100.8)	(180)	(0)	(25.9)
*trans-perp*	**1.190**	**1.450**	**1.440**	**0.980**	**111.2**	**105.4**	**100.7**	**–179.5**	**105**	**13.4**
	(1.188)	(1.481)	(1.446)	(0.978)	(108.4)	(104.7)	(100.1)	(176.6)	(101.0)	(12.1)
trans–trans	**1.190**	**1.450**	**1.440**	**0.980**	**111.2**	**105.3**	**100.6**	**179.7**	**180**	**21.0**
	(1.192)	(1.452)	(1.471)	(0.979)	(108.5)	(102.3)	(96.8)	(180)	(180)	(22.6)

a Results from our classical molecular mechanical model (bold) compared to *ab initio* data from the literature (within brackets).^37^

### 3.7 Nitric Acid

The molecular mechanical model for nitric acid (HNO_3_) was formulated using the previously established atom types OP, OQ, and NQ. To accurately reproduce the pure liquid properties of HNO_3_, fine-tuning of the OP-OQ interaction proved essential. An additional *C*_12_ LJ parameter was assigned to the OQ atom type (Table 2), specifically for its pairing with OP (Table 5). This adjustment was limited to the OP–OQ interaction, leaving the parametrization of prior species unaffected. Following refinement of the LJ parameters and optimization of the partial charges, the model delivered pure liquid properties (Table 6) and hydration free energy (Table 7) in close agreement with experimental data. To maintain the planarity of the HNO_3_ molecule, a high-barrier RB dihedral potential^41^ was combined with an improper dihedral term (Table 9).^42,43^

### 3.8 Nitrous Acid

The parametrization of nitrous acid (HNO_2_) followed a strategy akin to that of previous species, employing the established atom types OP, OQ, and NQ. However, the presence of two stable conformers, *cis*- and *trans*-HNO_2_, required additional considerations. These conformers, which are separated by a substantial energy barrier,^44–46^ collectively contribute to the experimentally determined hydration free energy of HNO_2_. Complete sampling of the conformational space via standard TI simulations would demand impractically long time scales. Enhanced sampling techniques have proven effective in analogous cases,^47^ but we opted for a simpler, thermodynamically equivalent approach. A thermodynamic cycle was established to link together the hydration free energies of individual conformers (Δ*G*_w,*trans*_ and Δ*G*_w,*cis*_) and the *trans*-to-*cis* isomerization free energy of HNO_2_ in the gas phase (Δ*G*_i_).^8^ This resulted in the following expression for the hydration free energy:

11 $$\Delta G_{w}=\Delta G_{w,trans}-RT\ln\frac{1+\exp\left[\left(-\Delta G_{i}+\Delta G_{w,trans}-\Delta G_{w,cis}\right)/RT\right]}{1+\exp\left(-\Delta G_{i}/RT\right)}$$

The Δ*G*_*i*_ term was directly derived from the O–N–O–H torsional potential (Table 9). The hydration terms, Δ*G*_w,*trans*_ and Δ*G*_w,*cis*_, were obtained from TI simulations (Table 7). In fewer than 1% of TI intermediate steps, isomerization occurred, requiring simulations to be restarted to ensure exclusive sampling of the intended conformational state. The resulting weighted average of the hydration free energies from both conformers produced an overall hydration free energy in close agreement with experimental data (Table 7).

### 3.9 Nitrogen Oxyanions

Molecular mechanical models for the nitrate (NO_3_^–^) and nitrite (NO_2_^–^) anions were developed using standard GROMOS atom types. Oxygen atoms were represented by the negatively charged OM type, while nitrogen was assigned the NR type, commonly used for planar fragments (Table 9). These atom types, with their notably large van der Waals radii, proved advantageous for reproducing the poorly structured hydration shell around NO_3_^–^.^48–52^ Partial charges were assigned respecting molecular symmetry and were empirically optimized for a correct description of hydration structure and ion pairing in solution. It is worth noting that the charge distributions in the final molecular mechanical models for NO_2_^–^ and NO_3_^–^ (Table 9) differed significantly from initial electronic structure calculations (Table 10). This discrepancy likely arose from model limitations, including the absence of polarization, the rigid structure of solvating water molecules, and the use of atom-centered point charges that fail to fully capture electron cloud delocalization. As shown in Figure 4, the water distribution around the NO_3_^–^ model closely aligned with more fundamental *ab initio* MD data from the literature, representing a clear improvement over earlier fully classical models.^24^ By contrast, the NO_2_^–^ model displayed a hydration shell that was slightly more structured than *ab initio* predictions.^25^ Nevertheless, it still reproduced a qualitative trend of *ab initio* simulations, namely that hydrogen bonds form predominantly between oxygen atoms and water, with fewer solvent molecules coordinating to nitrogen.^53^ Highly concentrated NaNO_3_ and NaNO_2_ solutions remained stable, reflecting the exceptionally high aqueous solubilities of these salts.^14^ Table 12 shows that solution densities closely matched experimental data. As illustrated in Figure 5, ionic interactions were well-balanced and led to ion pairing tendencies in close agreement with experimental observations up to concentrations of ∼4 mol/L.

**Figure 4:**
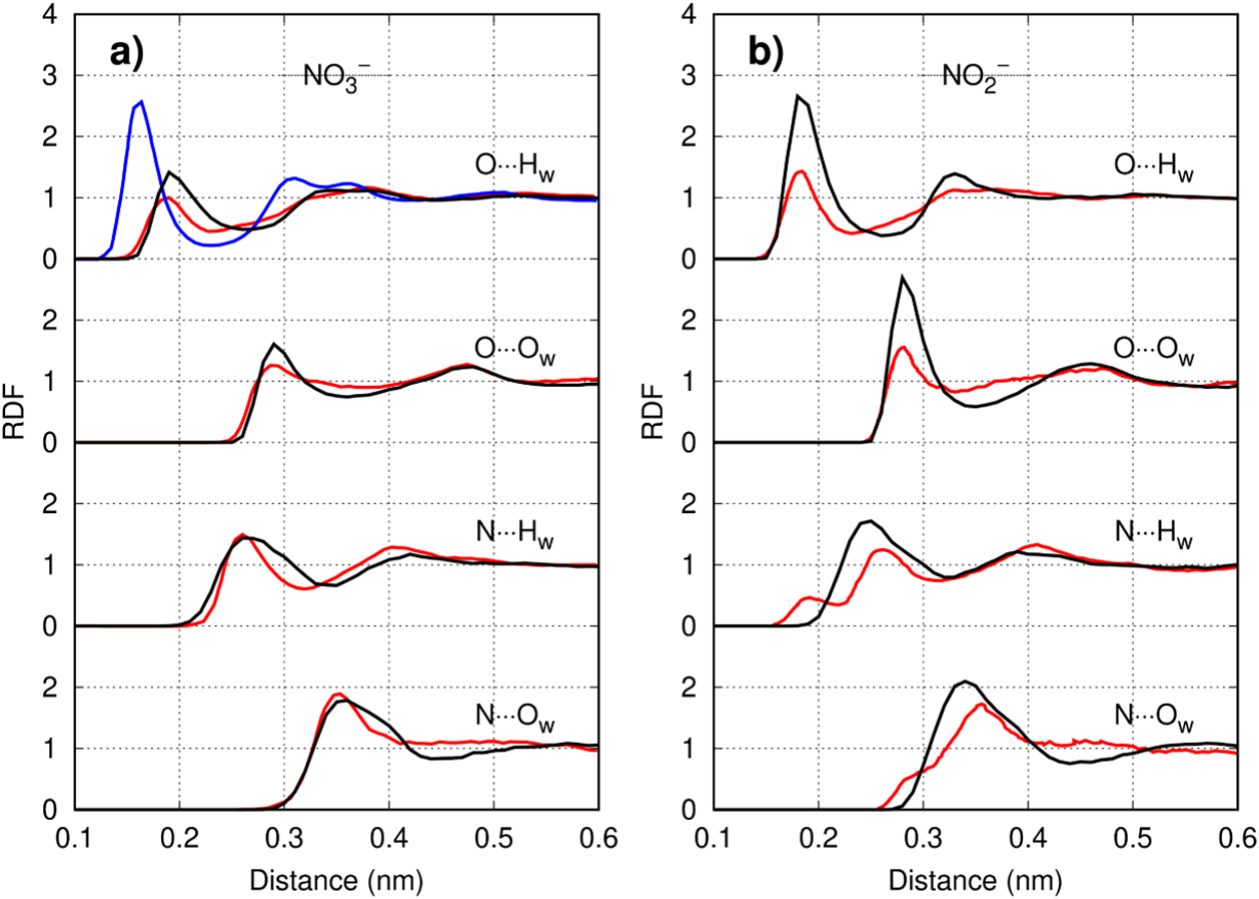
Hydration structure of (a) NO_3_^–^ and (b) NO_2_^–^ at infinite dilution. Radial distribution functions obtained from the classical GROMOS-RONS force field (black) are compared to *ab initio* (red) and classical (blue) simulations from the literature.^24,25^

**Figure 5:**
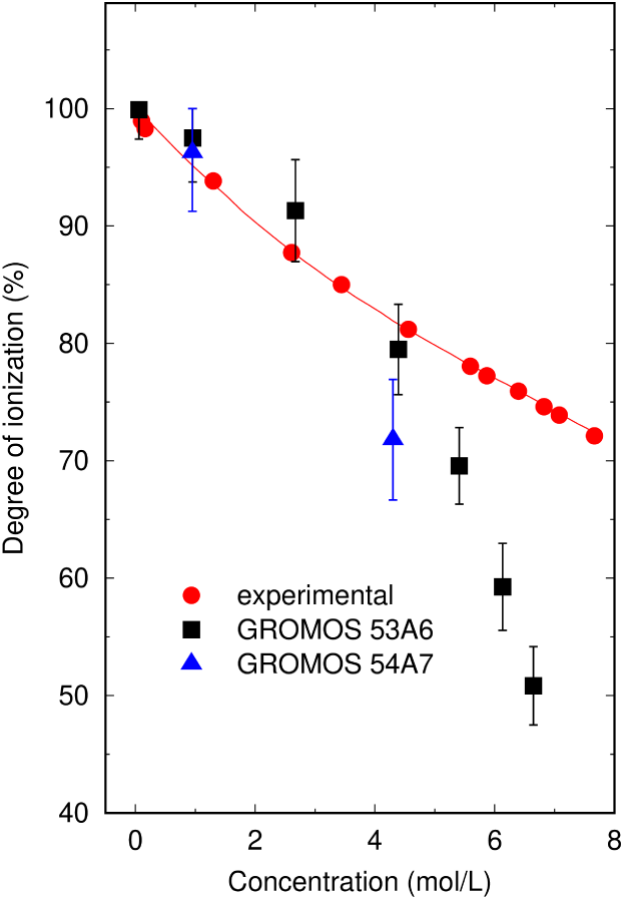
Ion pairing in NaNO_3_ aqueous solutions. Degrees of ionization at various concentrations, as obtained within the frameworks of GROMOS 53A6 and 54A7, are compared to experimental data from the literature.^30^

**Table 12:** Solution Properties of Nitrogen Oxyanions

		ρ (g/cm^3^)	
Salt	Concentration (mol/L)	This work	Reference data^a^
NaNO_3_	4.4	1.20	1.21
NaNO_2_	4.7	1.22	1.21

a From experimental measurements.^29^

### 3.10 Superoxide and Peroxynitrite

A model for the superoxide radical anion (·O_2_^–^) was developed using the negatively charged OM atom type, previously validated for nitrogen oxyanions. As a side note, initial attempts with the OO atom type resulted in excessive ion clustering and unstable ionic solutions.^4^ Similar to O_2_, the ·O_2_^–^ model offers limited flexibility in terms of adjustable parameters. As per symmetry, each oxygen atom necessarily carries half the total molecular charge. As depicted in Figure 6a, the OM-based model yielded stable solutions. However, more detailed analysis of the RDFs in Figure 7 reveals stronger hydrogen bonding with water in comparison to *ab initio* MD data from the literature.^26^ One possible refinement could involve introducing virtual interaction sites (e.g., along the interatomic bond axis) to better distribute the charge and mimic delocalization effects. For this study, however, we adhered to the standard GROMOS framework of atom-centered partial charges. The peroxynitrite anion (ONOO^–^) model was adapted from the existing HOONO parameters. The deprotonated oxygen was assigned the OM type, as it bears most of the anionic charge. Atom-centered partial charges were derived from electronic structure calculations (Table 10) and averaged across the *cis* and *trans* conformers of ONOO^–^ (Table 9). *Ab initio* data from the literature indicate that the *trans* conformer is higher in energy than the *cis* by ∼13–14 kJ/mol in the gas phase, and both are separated by a barrier of ∼102–106 kJ/mol.^54^ The O–O–N–O dihedral potential was adjusted to yield a similar behavior, as shown in Figure 8. This model also produced stable solutions (Figure 6b). As shown in Figure 9, the deprotonated oxygen atom acted as a strong hydrogen-bond acceptor, while the terminal oxygen at the opposite end had a poorly structured solvation shell. This behavior is in close proximity to *ab initio* data from the literature.^27,28^

**Figure 6:**
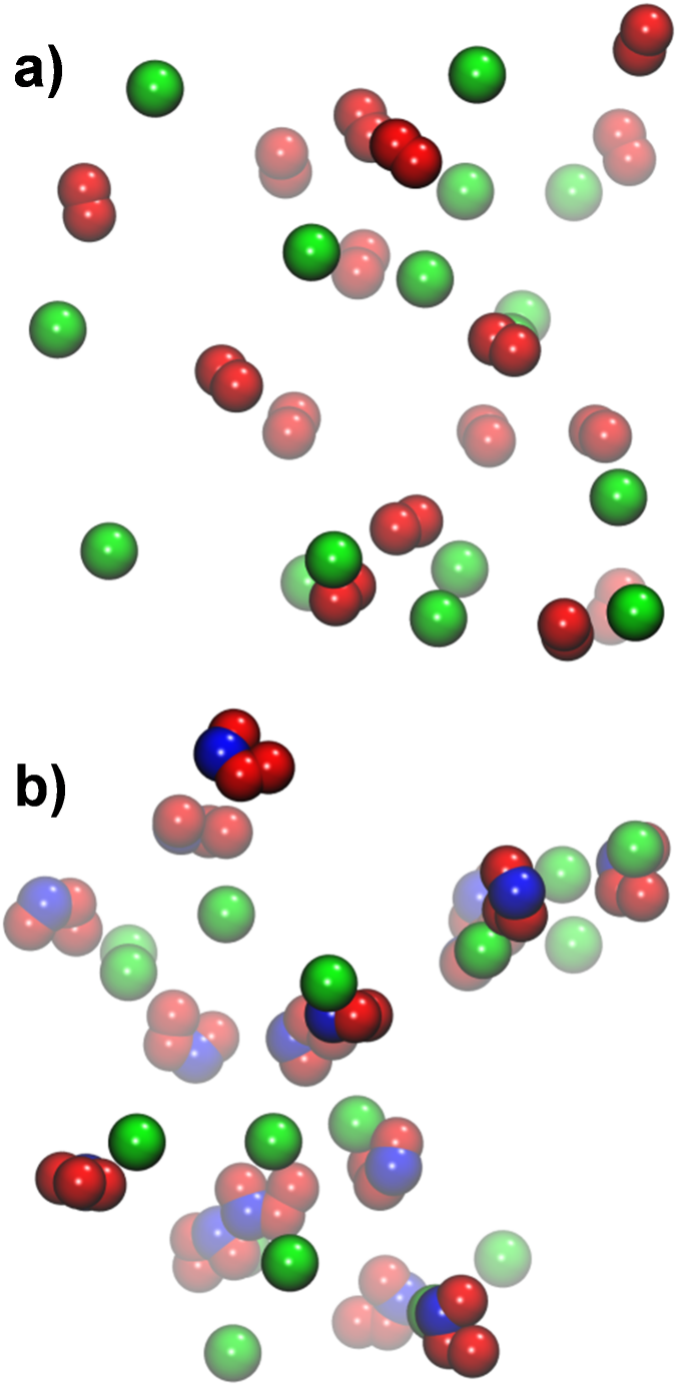
Images of (a) ·O_2_^–^ and (b) ONOO^–^ solutions after equilibration at ∼1 mol/L with Na^+^ counterions. Color code: red O, blue N and green Na^+^. Water molecules were omitted for clarity.

**Figure 7:**
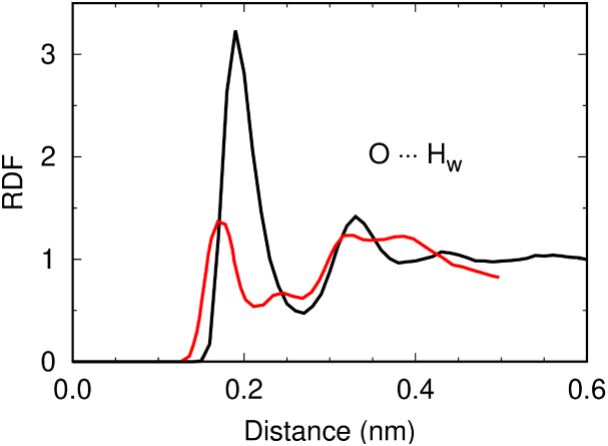
Hydration structure of ·O_2_^–^ at infinite dilution. Radial distribution functions obtained from from the classical GROMOS-RONS force field (black) are compared to *ab initio* (red) simulations from the literature.^26^

**Figure 8:**
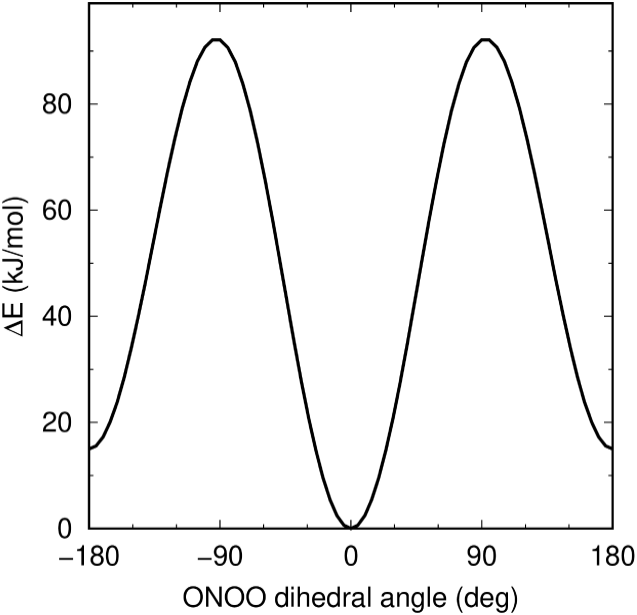
Dihedral energy profile of ONOO^–^ in vacuum according to the classical GROMOS-RONS force field.

**Figure 9:**
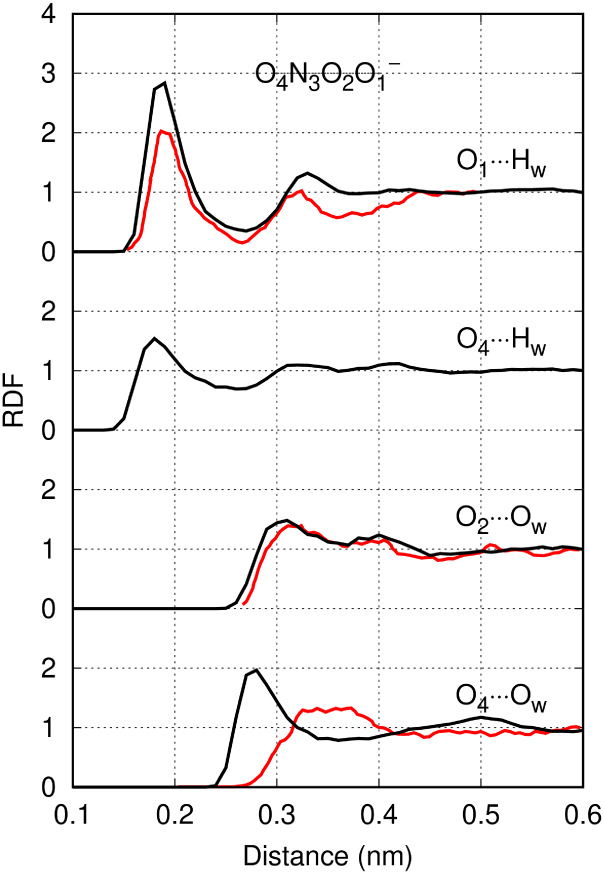
Hydration structure of *cis*-ONOO^–^ at infinite dilution. Radial distribution functions from the classical GROMOS-RONS force field (black) are compared to *ab initio* (red) simulations from the literature.^27,28^ Dimensionless *ab initio* RDFs were scaled based on the plateau region’s value.

### 3.11 Combination with Phospholipid Models

To enable MD simulations of RONS in the phospholipid membrane environment, LJ interactions were extended to include the atom types OML and CH3L, developed by Poger et al. within the context of a GROMOS-compatible phospholipid force field (Table 5).^55,56^

### 3.12 Extension to GROMOS 54A7

The GROMOS-RONS force field has been developed thus far within the GROMOS 53A6 framework. Adapting it to GROMOS 54A7,^57^ a version optimized for biomolecular simulations of proteins and lipids, is straightforward. The lipid atom types from Poger et al. have already been incorporated into the pair interaction tables of GROMOS 54A7, though with different names. The LJ parameters for Na^+^ and Cl^–^ ions were updated in GROMOS 54A7 for improved solvation properties, requiring adjustments to their interactions with our newly developed atom types. A separate force field file for use with GROMOS 54A7 is provided as supplementary data. As shown in Figure 5, the ionic interactions of nitrogen oxyanions remained well represented within the GROMOS 54A7 framework.

## 4 Discussion

The GROMOS-RONS force field provides a versatile tool for exploring the behavior of RONS across diverse systems, particularly where solvation and partitioning phenomena are key. The solvation properties obtained from our model RONS in water and hydrocarbons are summarized in Figure 10, showing good alignment with reference data. This force field supports a range of biomolecular applications, a few of which are highlighted here.

**Figure 10:**
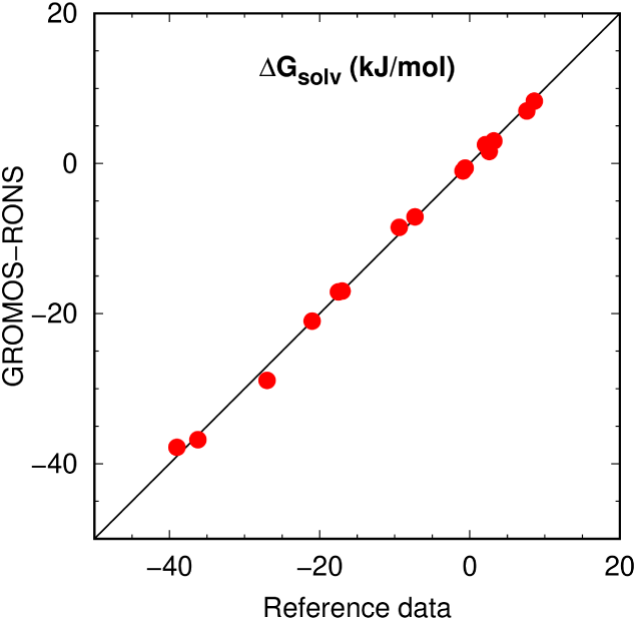
Overview of simulated (with the GROMOS-RONS force field) versus reference solvation free energies for RONS, with the diagonal line marking perfect agreement. Detailed results are provided in Tables 7 and 8.

Molecular mechanical models of small hydrophobic gases, such as O_2_ and ·NO, are valuable for studying lipid membrane permeation and pathways to protein binding sites. The transition from water to less hydrophilic environments demands a balanced representation of interactions in both media. The parameters proposed here have been included in a comparative survey of O_2_ models,^58,59^ confirming their reliable performance in capturing hydrophilic/hydrophobic partitioning, consistent with their design intent. The model did not perform well in capturing the enthalpy of vaporization of liquid O_2_, a thermodynamic state typically beyond the scope of most biomolecular simulations. Using the molecular models developed here, MD simulations revealed detailed information on O_2_ and ·NO distribution across phospholipid membranes.^4,6^ For O_2_, in particular, reasonable agreement with experimental data was found for the water/membrane partition coefficients across a broad temperature range, encompassing both gel and fluid phases of the membrane.^60^ Similarly, membrane simulations of the oxyradicals HO_2_· and HO· revealed that, although they reside near phospholipid headgroups, membrane fluidity and disorder allow them to reach double-bond sites along lipid acyl chains, where oxidative attack is expected to occur.^4^ The dynamics of ONOOH at membrane interfaces further showcases the model’s applicability. Simulations revealed a tendency for ONOOH binding and enrichment at the membrane-water interface.^5^ Equilibrated ONOOH molecules were cleaved into HO· and ·NO_2_ radicals in order to investigate their fate after a putative ONOOH homolysis reaction. A rapid separation was revealed: ·NO_2_ radicals favored the membrane interior, while some HO· radicals escaped to the aqueous phase, though others lingered near headgroups for extended periods. This behavior hints at localized reactivity and offers a potential explanation for enhanced intramembrane nitration.^61–63^ Another application lies in aquaporin-mediated transport. These channels, critical for water movement across membranes, also facilitate RONS passage, influencing redox signaling and oxidative stress responses in living organisms.^64^ Simulations confirmed the ability of aquaporins to transport H_2_O_2_ and possibly oxyradicals too.^7^ Aquaporin overexpression in cancer cells could at least partly explain their pronounced sensitivity to therapies based on RONS generation.^65^ Beyond biological applications, the model extends to atmospheric chemistry. The enrichment or depletion of RONS and other molecules at the water/air interface, key to understanding aerosol chemistry, lies well within its capabilities.^8^

Finally, all these examples underscore the adaptability of the GROMOS-RONS force field. As represented in Figure 1, a considerable number of different RONS have been parametrized, though this is far from an exhaustive list. Extension to other species should be straightforward following the protocol outlined here.

## 5 Conclusions

We developed the GROMOS-RONS force field, a collection of molecular mechanical models of a diverse array of RONS and related substances within the GROMOS 53A6 and 54A7 force field frameworks. By combining electronic structure calculations, thermodynamic integration, and equilibrium MD simulations, we established a comprehensive and validated parameter set. For electrically neutral species, this force field accurately reproduced hydration free energies; for small hydrophobic gases, it effectively captured hydrophilic/hydrophobic partitioning tendencies. For ionic species, the parameters yielded hydration structures consistent with *ab initio* MD simulations from the literature and exhibited well-balanced ion-pairing behavior. Validation against experimental benchmarks confirmed the reliability of the force field. GROMOS-RONS leverages the computational efficiency and established accuracy of the GROMOS force field family for biomolecular and condensed-phase simulations. This does not preclude RONS parametrization within other force field families. Provided as a well-documented reference with supporting files, the GROMOS-RONS force field lays a foundation for future studies, enabling extensions to additional species and deepening our understanding of RONS behavior across different scientific domains.

## Supplementary Material



## Notes

Force field and molecular topology files for RONS according to GROMOS 53A6 and 54A7 (ZIP)
